# Caregiver Networks of Older Adults With Alzheimer Disease: Design and Protocol for a Multisite Study Using Network Canvas

**DOI:** 10.2196/98270

**Published:** 2026-07-13

**Authors:** Tom Wolff, Celie Joblin, Kate Banner, Alaine Murawski, Patrick Janulis, Vanessa Ramirez-Zohfeld, Charlie Olvera, Lee Lindquist, Michelle Birkett

**Affiliations:** 1 Center for Computational and Social Sciences in Medicine, Feinberg School of Medicine, Northwestern University, 625 Michigan Avenue, Chicago, IL, 60611, United States, 1 2039930680; 2 Division of Geriatrics, Feinberg School of Medicine, Northwestern University, Chicago, IL, United States

**Keywords:** Alzheimer disease, age-related memory disorders, caregivers, social networks, personal networks, software

## Abstract

**Background:**

Patients with Alzheimer disease commonly rely on family caregivers for daily functioning. Research shows that relationships between caregivers and persons with memory loss have important effects on the health and well-being of both caregivers and persons with memory loss. However, most studies rely on a single caregiver–person with memory loss dyad as the unit of analysis, thereby neglecting the broader network of caregivers who collectively shape care experiences and outcomes.

**Objective:**

This study develops a conceptual and methodological framework for studying caregiver networks and investigates how the properties of caregiver networks relate to health and well-being outcomes for both persons with memory loss and caregivers. It measures and maps the social networks of caregivers of persons with memory loss while examining population heterogeneity in caregiver relationships and identifying network-based predictors of well-being for both caregivers and persons with memory loss.

**Methods:**

Our team is conducting a large multilocation (Illinois, Indiana, and Hawaii) study comprising 200 caregiver networks of persons with memory loss, supported by data from persons with memory loss and their caregivers. Networks will be collected using cutting-edge Network Canvas software tools, developed by members of our team within the Complex Data Collective, and analyzed using both quantitative and mixed methods.

**Results:**

This study was funded in August 2023 by the National Institutes of Health (R01AG083034), with an expected end date of July 2028. This study protocol was approved by the institutional review board of Northwestern University (STU00219675) and was piloted internally before participant recruitment began in January 2025. As of March 2026, a total of 155 participants have been enrolled. Enrollment is planned to end by April 2027, with results expected between August 2027 and November 2027.

**Conclusions:**

This study advances research on caregivers of persons with memory loss by operationalizing a scalable and replicable approach to measuring caregiver networks. The ability to easily measure and identify structural features of caregiver systems helps in identifying predictors of caregiver experiences and outcomes and provides large benefits for the caregiver–person with memory loss research community.

## Introduction

### Caregiver Networks for Persons With Memory Loss

Alzheimer disease (AD) affects more than 6.2 million Americans aged 65 years and older, and this number could grow to 13.8 million by 2060 [1-4]. Over time, older persons with memory loss experience worsening memory and rely on caregivers or supporters to function on a daily basis [5-8]. In 2020, more than 11 million family members and other unpaid caregivers provided an estimated 15.3 billion hours of care to people with AD and other dementias [1,9]. Approximately, US $216 billion worth of services were provided by unpaid or informal caregivers, averaging 22 hours per week [10,11].

Research on informal caregivers tends to focus on family caregivers, which by definition include spouses, children, parents, relatives, or loved ones who provide assistance that is above what is typically expected in that relationship [12]. Evidence shows that most family caregivers are ill-prepared for their caregiving role and that caregivers often experience a sense of burden from their respective roles [13-22]. This feeling of burden is associated with declines in the well-being, mental health, and health-related quality of life of caregivers [18-22]. Caregiver burden also affects patient health care, and increased burden has been shown to negatively impact patients’ emotional outcomes, interfere with how well caregivers assess patient pain and other symptoms, and influence long-term medical decision-making [23-28].

In studying the relationship between persons with memory loss and caregivers, AD research traditionally focuses on the “caregiver-receiver dyad” as its unit of analysis [29,30]. The caregiver-receiver dyad is made up of 2 people, with only a single caregiver providing care to a person with memory loss. Here, the care recipient (ie, the person with memory loss) is viewed as a potential stressor, and the caregiver is assessed in terms of individual outcomes (eg, depression and health) [31]. Although commonplace, this perspective overlooks the fact that there is significant population heterogeneity in how persons with memory loss define “family” caregivers, both in terms of the quantity and types of care provided. This leads to “missed opportunities” in recognizing the contributions of multiple family members who simultaneously occupy vital caregiver roles and whose perspectives might jointly inform patient care. In reality, persons with memory loss are supported by multiple family members, partners, and friends who form a *caregiver network* that is largely ignored in existing research [32-34].

The idea of caregiver networks is not only intuitive but also consistent with popular perspectives for contextualizing public health. In particular, it aligns well with social ecological model frameworks that understand health behaviors and outcomes as being shaped at 4 different levels: individual, interpersonal, community-based, and societal. In such a framework, networks represent the interpersonal level at its core, accounting not only for the effects of single caregiver–person with memory loss relationships but also for a combined set of relationships simultaneously acting upon one another [35]. Social network data and analysis methods offer a better understanding of the social relational contexts that individuals inhabit and highlight both the protective and risky aspects of their connections to one another [36-38]. Beyond theory, caregiver networks are equally relevant to clinical practice. The ultimate goal of person with memory loss–caregiver research and family-framed dementia care is for health care providers to know and understand persons with memory loss and family caregivers within the context of their relationships in order to develop a plan of care that meets the biopsychosocial needs of the person with memory loss. Providers taking this approach aspire to consider the needs, wishes, and resources of caregivers so that such a plan is feasible, likely to be implemented, and will promote the safety and well-being of all involved supporters.

Our team has been at the forefront of efforts to draw attention to caregiver networks in AD research. In a pilot study, members of our team surveyed a national cohort of 97 family caregivers using open-ended questions to inquire about their experiences with social support networks [39]. Results from a constant comparative qualitative analysis of responses confirmed the notion that persons with memory loss receive care from multiple caregivers rather than a single caregiver-receiver dyad. Furthermore, results provide evidence that caregiving networks have different compositional characteristics (eg, larger or smaller size, higher proportions of kin and female caregivers, and proximity of caregivers) associated with more hours of care provided and varying network structures [40]. Although this work highlights the existence of caregiver networks, additional work and data are needed to fully understand how these networks shape the experiences and outcomes of caregivers and persons with memory loss.

### Positive and Negative Aspects of Caregiver Networks

The importance of social networks to people’s health is known across a variety of contexts, with previous work showing the many ways in which networks can be beneficial or detrimental to people’s health. Although underexplored in AD research, social networks are expected to have similar effects on persons with memory loss and their caregivers [41-43]. Relationships between caregivers can lead to positive interactions (eg, an out-of-town sister providing care so that the primary caregiver can go on a vacation) or negative interactions (eg, conflicts or disagreements) that affect an individual caregiver’s well-being [44]. In some cases, families of persons with memory loss may impose responsibilities on a single primary caregiver for a long period of time in a way that creates conflict and resentment within the family [45]. Conflict among family members regarding perceived inequities in the distribution of caregiving tasks has been identified as a major cause of caregiver distress [46]. Additionally, a large number of family members involved in caregiving can create coordination problems that may themselves lead to conflict [28,42].

Indeed, members of our team have made initial strides in identifying sources of conflict in caregiver networks and developing strategies to address them. This work has revealed that many older adults believe their families will handle any issues in the event they develop AD, yet few, if any, explicitly discuss these potential issues with their loved ones [47]. This results in conflicts between family members and persons with memory loss when AD emerges, as well as among family members over opinions on day-to-day and long-term care [48]. In response to these findings, members of our team developed an online planning tool (PlanYourLifespan.org) to help older adults and their families in discussing and planning for aging-in-place and long-term care needs in the event of worsening AD [49-51]. Other members of our team have adapted existing tools, such as Negotiations and Dispute Resolution (NDR) training and Life Enhancing Activities for Family Caregivers (LEAF), to help resolve family caregiver conflicts and improve caregiver well-being [52-62]. Despite the promise of these interventions in alleviating caregiver conflicts, much remains to be learned about the underlying causes of these conflicts within caregiver networks. Moreover, the potential positive effects of caregiver networks on the well-being of caregivers and persons with memory loss remain understudied.

### Measuring Caregiver Networks Using Network Canvas

A precursor to studying caregiver networks is the development of adequate methods for capturing them in surveys and interviews. Self-reported social network analysis studies are often complex and challenging, both during the interview process and when conducting data management following interviews. Historically, social network data have been collected through in-person, pen-and-paper-based interviews, a process that is burdensome, prohibitively slow, and resource-intensive [63]. Network data captured using these methods require extensive manipulation in order to be usable for analysis, creating a major barrier for those unfamiliar with graph-based data structures. These challenges are exacerbated when attempting to capture “complex” network data involving multiple types of individuals or relationships across multiple time points. Consequently, network data capture is often inaccessible to researchers who lack substantial training in network science, extensive resources, or the expertise to create bespoke software or data management solutions [64,65].

In response to these difficulties, members of our team developed the *Network Canvas* software suite with support from the National Institute on Drug Abuse (R01DA042711, R34DA052216-S1, and R01DA057973; principal investigator is MB) [63]. Network Canvas is a set of complementary tools designed to simplify the collection and storage of complex social network data, with an emphasis on usability and accessibility across platforms and devices. It is specialized for capturing contextual and social network data using touch-optimized interfaces. Most self-report survey tools are optimized for individual-level data capture and are thus not optimized for network data capture, which often entails asking study participants to answer the same questions over multiple entities (eg, individual people and caregivers). Network Canvas is specifically designed to address this shortcoming, using a unique experience that prioritizes a visual approach, tactility, microinteractions, and physical metaphor to enable study participants to interact with their own network [66]. This tool produces a more intuitive interview process for participants, minimizing response burden and attrition during data collection. For researchers, Network Canvas offers a streamlined research workflow. Users can easily build their own network interview protocols using a graphical user interface, deploy these protocols across a variety of in-person contexts, and export collected data in formats that require far less reshaping than those collected using conventional surveys.

To date, Network Canvas has been used in at least 63 National Institutes of Health (NIH)–funded studies and has demonstrated higher-quality data collection compared to other survey tools. It has drawn strong enthusiasm among social network scholars generally, with members of our team having been invited to share Network Canvas in workshops and presentations at numerous universities and the NIH. Network Canvas also received the 2025 William D. Richards “Lifetime Achievement” Software Award from the International Network for Social Network Analysis. The success of the software speaks to its accessibility and applicability to diverse populations and settings, which continues to grow with time. A recent partnership with members of our team involved the development of a specific “dyad census” interface designed for use by patients with AD (R01AG057739), highlighting its value for studying caregiver networks.

### Objectives

In this paper, we outline the method for capturing and studying caregiver networks of persons with memory loss. The overall goal of this project is to use social network measurement software (ie, Network Canvas) to capture expanded definitions of “family” caregivers of people with AD and AD-related dementias (ADRD), with a special focus on positive and negative relationship aspects. Through a multisite deployment across 3 US states (Illinois, Indiana, and Hawaii), we aim to survey the networks of 200 persons with memory loss through interviews with persons with memory loss and/or their caregivers, including the forms of support provided within caregiver networks and the positive and negative experiences associated with these networks. Furthermore, using mixed methods, we examine the heterogeneity of relationships within and between networks and assess how caregiver networks shape the well-being of caregivers and persons with memory loss. Our efforts are guided by 2 primary aims.

The first aim is to measure and map the social network of caregivers of persons with memory loss using the Network Canvas methodology and examine the population heterogeneity in how individuals define “family” caregivers. We will use Network Canvas to capture participants’ caregiver networks, quantifying the number of caregivers supporting individual people with memory loss and the relationships that exist between caregivers. Our ability to collect these data and produce network-based measurements will be used to test a substantive primary hypothesis and a more exploratory methodological hypothesis:

- H1: Caregiver–person with memory loss dyads are not reflective of the real-world heterogeneous experiences of persons with memory loss. Caregiver networks offer a better framework for research focused on the care of persons with memory loss.
- H2: Network Canvas will be an effective tool for mapping persons with memory loss–caregiver networks.

The second aim is to measure and determine network predictors of positive well-being among caregivers and persons with memory loss. Using measures developed in pursuit of aim 1 and other measures derived from Network Canvas data, we will determine whether network-based factors contribute to individual-level health outcomes. To address this, we will use both quantitative and qualitative analyses to evaluate the following primary hypothesis (H3) and exploratory hypothesis (H4):

- H3: Caregiver support that persons with memory loss receive from their network will be heterogeneous (eg, multiple caregivers providing small amounts of care and a primary caregiver providing the bulk of care with smaller support from others).

- H4: Negative intranetwork influences (eg, conflict, differing expectations) and positive intranetwork influences (eg, teamwork, communication) will affect the burden, anxiety, stress, and well-being of caregivers.

Through this research, we plan to (1) develop a better understanding of how to measure caregiver networks; (2) identify which aspects of caregiver networks are most predictive of psychological and physical well-being of caregivers and persons with memory loss; and (3) operationalize a network measurement approach for caregiver systems that can be shared with other researchers, providing a larger benefit for the caregiver and person with memory loss research community.

## Methods

### Overview of Study Design

This study plans to collect data on caregiver networks consisting of 200 unique persons with memory loss networks based on information collected from persons with memory loss and/or caregiver participants. We will recruit persons with memory loss at the core of these networks through several channels, including our team’s established community partnerships, physician clinics, and NIH-funded cohorts. Data collection will occur primarily across 3 different US states, which will be able to enroll and interview participants in person, in addition to completing data collection virtually, ensuring adequate representation of minority populations and allowing this study to better ascertain the potential differences in caregiver network size and structure between different groups and locales. We will use snowball sampling recruitment to enroll 200 persons with memory loss and collect data from up to 800 members of these participants’ networks [67]. Persons with memory loss networks may be reported by an individual (caregiver or person with memory loss only) or multiple individuals (eg, 1 caregiver and 1 person with memory loss or only caregivers). To the extent that they are capable, all sampled participants will complete mixed methods interviews capturing their personal information, their knowledge of relationships comprising caregiver networks, qualities of these relationships, and patient-centered outcomes for relevant persons with memory loss. The interviews will combine established methods and measures for studying persons with memory loss using *Network Canvas*—a cutting-edge tool for network data capture developed by our team—to create an optimal instrument for studying patients, caregivers, and their networks at different levels. This instrument will capture individual, interpersonal, community, and societal factors shaping the experiences of persons with memory loss and their caregivers, consistent with a social ecological framework.

In pursuit of aim 1, our team will use data collected from the Network Canvas portion of our interviews to describe the size and structure of caregiver networks. Using simple quantitative methods, we will show that many, if not most, persons with memory loss receive care from more than a single person, demonstrating the need to pay attention to caregiver networks over single caregiver–person with memory loss dyads. Furthermore, data used in aim 1 will show the ability of Network Canvas to capture and map caregiver networks and its use for future studies of patients with ADRD. Consistent with our approach to data collection, analyses in pursuit of aim 2 will take a mixed methods approach to identify predictors of persons with memory loss and caregiver well-being. Quantitative and qualitative analysis of study data will highlight the role of individual, network, social, and cultural differences on caregiver burden, anxiety, stress, and other outcomes.

### Target Sample

This study aims to capture a diverse, multistate, representative sample of caregiver networks comprising 200 persons with memory loss through interviews with both persons with memory loss and/or their caregivers. To determine the target sample size, we conducted a statistical power analysis, which identified a size of about 160 networks as being adequate to detect differences in key quantitative outcome measures [68,69]. Accordingly, a larger sample size is expected to provide adequate power for the quantitative analyses. For the qualitative analyses, previous work suggests that a sample of 12 participants is sufficient to reach thematic saturation in this population of interest. As such, we anticipate that our sample of 200 caregiver networks will afford us not only basic thematic saturation but also the ability to describe differences between persons with memory loss with regard to cognitive levels, networks, gender and sexual orientation, urbanicity, and race or ethnicity [70-72]. Efforts to enroll a representative sample with regard to demographic attributes will be guided by a multipronged recruitment strategy across multiple US states, as well as a concerted effort to recruit equal numbers of White and non-White persons with memory loss. Recruitment and data collection will occur during years 2 to 4 of the study.

### Recruitment

#### Eligibility

Participants in this study must meet different eligibility criteria depending on whether they are persons with memory loss or caregivers. Participants with memory loss must be aged 65 years or older, speak English, and have a score of at least 2 on the Ascertain Dementia 8-item Informant Questionnaire (AD8TM), a value indicating likely cognitive impairment [73]. During the screening process, potential participants with memory loss will complete the validated University of California, San Diego Brief Assessment of Capacity to Consent (UBACC) to determine their ability to provide informed consent. Persons with memory loss who are not deemed capable of providing informed consent will be excluded from participation [74,75].

Caregiver participants must be at least 21 years of age, speak English, and provide some form of support (eg, emotional, social, physical, or task-related) to a person with memory loss aged 65 years or older who has a score of at least 2 on the AD8TM or to another adult caregiver. Caregivers must also indicate the ability to give informed consent, as assessed by the UBACC. In addition to these criteria, all potential participants must either have access to and enough familiarity with the internet to facilitate a Zoom call with research staff, or they must have the ability to meet with research staff in person, where available. These requirements ensure that research staff can successfully collect data from participants once they are deemed eligible.

#### Recruitment Activities

Over a period of 3 years, we will recruit both persons with memory loss and caregiver participants through a set of interdisciplinary community partners, physician clinics, and cohorts of participants from prior NIH-funded studies, each of which has established connections with members of our team. To improve the representativeness of our sample, we have partnered with organizations across 3 US states (Illinois, Indiana, and Hawaii).

This study will also perform targeted recruitment of participants from prior NIH-funded research cohorts involving similar research topics and/or populations of interest (R01AG068421 and R01AG058777; principal investigator is LL). Prior studies asked members of these cohorts if they would be willing to be contacted for additional research opportunities. Our team will invite those who indicated willingness to participate in this study through their shared contact information.

Following initial contact, potential participants will be given a phone number to call a research assistant to participate. Upon being called, the research assistant will obtain verbal consent to ask a brief series of questions to assess eligibility, including the UBACC (see *Eligibility* section). For participants who meet the conditions for eligibility, the research assistant will schedule a time at which the participant can complete the electronic consent. Upon consent, they will be given access to the data collection survey.

#### Recruitment of Caregivers From Person With Memory Loss Responses and Without Person With Memory Loss Participation

We expect to recruit caregiver participants through implicit referrals given by the persons with memory loss for whom they provide care. Participants will receive research study contact information at the end of the Network Canvas interview and will be asked to share this information with any caregivers and/or the person with memory loss they have identified during their study interview. If the fellow caregivers and/or person with memory loss are interested in participating, they will contact the study team directly and be screened for eligibility. We plan to interview as many as 4 caregivers or supporters for a given person with memory loss, allowing us to potentially capture multiple perspectives on individual caregiver networks.

In other instances, we expect to recruit caregiver participants directly, without any involvement from their respective persons with memory loss. We anticipate that most persons with memory loss contacting research staff for participation will be in the early stages of AD and that patients in the later stages of AD or ADRD may not have the ability to contact us or give consent to the study. To ensure the inclusion of persons with moderate-to-severe dementia, caregivers of persons with memory loss can also contact research staff directly for participation.

### Data Collection

#### Social Ecological Model Measures

Participants will complete surveys with the assistance of research staff, which will be conducted either via Zoom videoconferencing or in person, depending on the participant’s preference. The survey is designed to capture variables aligning with different levels of the social ecological model, with items capturing contextual factors at the individual, interpersonal (and network), community, and societal levels.

#### Individual Measures

For each individual, or “ego” in network parlance, we will ask demographic questions pertaining to age, educational attainment, occupation, sex at birth, current gender, sexual orientation, race, and ethnicity. The primary measures such as positive affect and well-being will be assessed using the Neuro-QoL (quality of life in neurological disorders) for all participants [76]. Measures of participant stress, fatigue, self-efficacy, and anxiety will also be collected from all participants using validated Patient-Reported Outcomes Measurement Information System (PROMIS) and NIH Toolbox inventories [77,78]. We plan to use the Neuro-QoL, the PROMIS, and the NIH Toolbox items as outcome measures of interest in exploratory analyses surrounding H4. The survey will take additional individual-level measurements depending on whether a participant is a person with memory loss or a caregiver. For participants with memory loss, we will assess their cognition using the Montreal Cognitive Assessment—Blind version [79,80]. For caregivers, we will collect self-reports of burden and anxiety using survey items specifically tailored for caregiver settings. Individual measures will primarily be captured using the Research Electronic Data Capture software suite (REDCap, Vanderbilt University) [81].

#### Interpersonal and Network Measures

**Figure 1.**
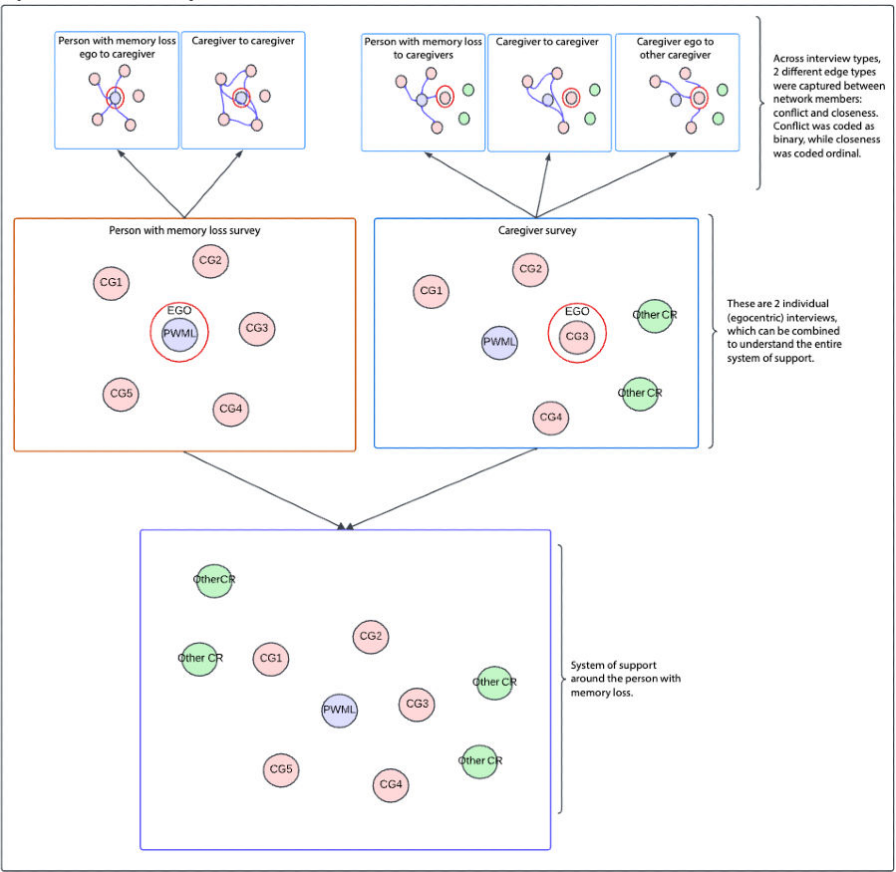
Network interview diagram. System of support around the persons with memory loss: What we are attempting to build through interviewing up to five separate individuals (the persons with memory loss who completes the “Persons with memory loss survey;” up to 4 individuals who complete the “Caregiver survey”) and consolidating their data. Person with memory loss: within each ”Caregiver survey“ completed by individuals within the system of support around the persons with memory loss, the person with memory loss is an “Alter.” Within the ”Persons with memory loss survey,” they will be an “Ego.” CG: They are a primary caregiver of the persons with memory loss. When they are the person interviewed by us within the ”Caregiver survey,” they are a ”CGEgo.” Within the ”Persons with memory loss survey,” they will be a “CGAlter.” Within the ”Caregiver survey” completed by other individuals within the ”system” around the persons with memory loss, they will also be a “CGAlter.” Other CR: these are individuals whom the CG also provides support to beyond the persons with memory loss. They are not the primary focus of this grant, but they are important to understand as they might constrain the ability of the CG to support the persons with memory loss. Ego: the person who completes an individual network canvas survey. Alter: A person who is named in a network canvas survey. CG: caregiver; CR: care recipient.

Two distinct network interviews were designed: one aimed at eliciting the networks of persons with memory loss and the other aimed at eliciting the networks of caregivers of persons with memory loss. Details of each of these protocols are described in Tables 1-3 and Figure 1. Interpersonal and network measures will be captured using interfaces built into our team’s Network Canvas software suite [63,82,83]. These interfaces correspond to 3 common stages of social network interviews: name generation, name interpreting, and edge (relationship) generation.

**Table 1.** Demographic attributes captured by node type across person with memory loss and caregiver interviews.

Node type (N)	Person with memory loss interview		Caregiver interview			
	Person with memory loss-ego (1)	Caregiver (1+)	Person with memory loss (1)	Caregiver-ego (1)	Caregiver (0+)	Other care recipients (0+)
Name	✓	✓	✓	✓	✓	✓
Age	✓	✓	✓	✓	✓	✓
Education	✓	✓	✓	✓	✓	
Employment status		✓		✓	✓	
Occupation		✓		✓	✓	
Sex	✓	✓	✓	✓	✓	✓
Gender	✓	✓	✓	✓	✓	
Sexual orientation	✓	✓	✓	✓	✓	
Race	✓	✓	✓	✓	✓	✓
Ethnicity	✓	✓	✓	✓	✓	✓
Marital status	✓	✓	✓	✓	✓	
Relationship^a^	✓	✓	✓	✓	✓	✓

a Relationship with focal person with memory loss, categorized as either spouse or partner, sibling, child, daughter or son-in-law, grandchild, niece or nephew, cousin, friend, neighbor, paid caregiver, or other.

**Table 2.** Relationship and caregiving attributes captured by node type across person with memory loss and caregiver interviews.

Node type (N)	Person with memory loss interview		Caregiver interview			
	Person with memory loss-ego (1)	Ego to caregiver	Person with memory loss (1)	Ego to people with memory loss	OCG^a^ to people with memory loss	Ego to OCR^b^
YearsKnown		✓		✓	✓	
LiveWith		✓		✓	✓	
RoutineFamiliarity		✓		✓	✓	
EgoCare				✓		
TypeCareReceived				✓		
TypeCareProvided						✓
DistanceFromPersonWithMemoryLoss		✓		✓	✓	
YearsSupport		✓		✓	✓	
SeeFrequency		✓		✓	✓	
TalkFrequency		✓		✓	✓	
HoursPerWeek^c^		✓	✓		✓	✓

a OCG: other caregiver.

b OCR: other care recipient.

c Hours per week that caregiver spends providing care to person with memory loss.

**Table 3.** Influence and support attributes captured by node type across person with memory loss and caregiver interviews.

Node type (N)	Person with memory loss interview		Caregiver interview			
	Person with memory loss-ego (1)	Caregiver hours on ego	Ego hours on person with memory loss	Caregiver-ego (1)	OCG^a^ hours on person with memory loss	Ego hours on OCR^b^
HealthDecisioninfluence			✓		✓	✓
DailyDecisioninfluence			✓		✓	✓
CareStrategyinfluence			✓		✓	✓
Financialinfluence			✓		✓	✓
Overallinfluence			✓		✓	✓
MostTrusted			✓		✓	✓
MajorCrisisHelp			✓		✓	✓

a OCG: other caregiver.

b OCR: other care recipient.

During the *name generation* stage, participants will be asked to name people in their social network, referred to as “alters” in network terminology. Persons with memory loss receive the prompt: “Please add anyone you can think of that provides you care or support (eg, physical, social, emotional, task-related),” while caregivers receive the prompt: “Who else provides care or support (eg, physical, social, emotional, task-related),” after first nominating the person with memory loss. Names given in response to this question by persons with memory loss will be used in snowball sampling to recruit additional caregiver participants (see section *Recruitment of Caregivers From Persons With Memory Loss Responses and Without Person With Memory Loss Participation*). Given the distinct roles in caregiver–person with memory loss relationships, each caregiver network collected will have a “focal person with memory loss” around which all relationships are centered. For participants with memory loss, this focal person with memory loss will be themselves. For caregivers recruited through a person with memory loss response, the focal person with memory loss will be the person with memory loss through whom they were recruited. Caregivers recruited without the involvement of a person with memory loss will identify a single focal person with memory loss during the name generation stage. Of note, caregiver interviews also included a name generation item about other care recipients—or individual caregivers “provided regular support (eg, physical, social, emotional, task-related) to” who were not the person with memory loss.

In the *name interpreting* stage, participants will be asked to state each named alter’s relationship with the focal person with memory loss and describe each alter’s demographic characteristics (age, sex, gender, sexuality, race or ethnicity, education, and employment status). They will also be asked to identify emotional aspects of each alter’s relationship with the focal person with memory loss, rate each alter’s emotional “closeness” with the focal person with memory loss, and identify whether the alter and the person with memory loss have conflict with each other [67,84]. Paired with data provided from the name generation stage, these measures will allow us to identify the *size* and *composition* of caregiver networks (see *Aim 1: Measuring and Mapping Caregiver Networks* section for details), as needed to validate our first primary hypothesis (H1). The name interpreting stage will include several measures capturing behaviors within person with memory loss–caregiver dyads, with participants estimating the frequency with which alters visit, communicate with, and provide support to the focal person with memory loss. Recognizing the many ways in which one can provide social support, participants will be asked multiple name interpreter questions that capture provisions of mobility, transportation, medical, and financial support. Measures pertaining to these forms of support will allow us to identify heterogeneity in support provided by caregivers within networks as we hypothesize in H3. Additionally, the name interpreting stage will ask participants to rate the level of influence that each alter has regarding overall and specific aspects of the care of a person with memory loss (daily decisions, health decisions, care strategy, and finances).

For the final *edge generation* stage, the survey will ask participants to identify whether pairs of alters in their caregiver network know one another. If 2 alters are known to each other, the survey will ask the participant to rate the emotional closeness of their relationship and whether the alters have conflict with each other. Alongside items collected in the name interpreting stage, edge generation responses will allow us to fully map the full set of relationships constituting caregiver networks and assess how positive and negative feelings arise throughout them, providing data on network *density* (see *Aim 1: Measuring and Mapping Caregiver Networks* section for details) to enable richer analyses pursuing H1. In past network interviews, the edge generation stage is often the most tedious, with participants having to answer repetitive survey items for each pair of named alters in a network. However, Network Canvas offers an interface that allows participants to effectively draw connections between alters in real time, considerably reducing the response burden of edge generation on participants.

Beyond name generation, name interpreting, and edge generation stages, the survey will provide participants with additional items for describing and identifying emotional processes in their networks. These include the NIH Toolbox inventory for social cohesion, as well as the Dutch Test for Conflict Handling, which assesses how participants handle conflicts across 5 different areas [85]. Participants will be asked open-ended questions about conflicts within their networks, allowing us to conduct qualitative analyses that may capture important details lost by standard survey methods. Finally, caregiver participants will be asked about feelings of positivity in their relationships through the Positive Aspects of Caregiving inventory, which identifies positive consequences of caregiving, such as feeling more useful, feeling appreciated, and strengthening relationships with others [86].

#### Community and Societal Measures

To capture community factors that extend beyond the immediate caregiver network, all participants will be given NIH Toolbox items pertaining to their perceived availability of friendships and neighborhood safety, as well as a PROMIS inventory used to identify perceptions of having social support. To assess even broader societal perceptions that may shape relationships between caregivers and persons with memory loss, participants will be provided with the Familialism questionnaire, which ascertains people’s beliefs about what obligations are owed to older adults. Finally, participants will be questioned about perceptions of societal stigma around being a caregiver and how they feel society reacts to their own roles as persons with memory loss or caregivers.

### Analysis

A combination of descriptive statistics, more detailed quantitative methods, and qualitative methods will be used to assess the study hypotheses. Specific analytic strategies for each study aim and hypothesis are detailed in the following sections.

#### Aim 1: Measuring and Mapping Caregiver Networks

This study’s initial aim is to measure and map caregiver networks of persons with memory loss in order to examine population heterogeneity in how individuals define “family” caregivers. To this end, we will calculate descriptive statistics on our collected network data to quantify the size, structure, and types of networks reported to us by participants. Quantitative measures will be calculated from Network Canvas data using the R programming language, and subsequent analyses will be conducted using a combination of R and Stata.

The primary hypothesis within this aim states that *caregiver–person with memory loss dyads are not reflective of real-world heterogeneous experiences of persons with memory loss, making a focus on caregiver networks a better approach for research focused on care for persons with memory loss* (H1). At the most basic level, we will test this hypothesis by counting the number of caregivers (and, by extension, person with memory loss–caregiver dyads) that participants report, which is the most basic indicator of caregiver network *size*. Distributions of network size, both for the overall sample and within subsets of our sample, will determine the extent to which persons with memory loss rely on caregiver networks for support—as opposed to singular dyads—and whether network size differs according to social, demographic, and regional attributes.

While network size is most directly applicable to testing H1, complementary measures will allow us to map caregiver networks in detail. Using data generated from the name interpreter stage of the network survey (see *Individual Measures* section for details), the *composition* of caregiver networks will be measured with regard to demographics (eg, caregiver age, race, sex, and gender) and the kinds of relationships caregivers have with persons with memory loss (eg, children, relatives, friends, neighbors, and paid caregivers). Data captured by the edge generation stage of the survey will allow us to trace relationships between caregivers in addition to person with memory loss–caregiver dyads. As a result, we will also measure the *density*, or overall connectivity, of caregiver networks to identify the extent to which members of caregiver networks know one another and have the capacity to coordinate care. Variations in density measures will further characterize caregiver networks with respect to specific qualities. Such variations include density based on the level of emotional closeness between individuals, the existence of conflict between individuals, and specific forms of social support given by caregivers to their focal persons with memory loss.

We anticipate that descriptive analyses of size, composition, density, and other network-based metrics will be sufficient to achieve our first study aim. However, we imagine that the final sample will be large enough to power partitioning methods such as hierarchical clustering and/or latent class analysis on our data. Following past work on personal networks, we anticipate that these methods will allow us to develop a typology of persons with memory loss–caregiver networks with respect to select properties, providing simple heuristics for recognizing different kinds of networks that caregivers and practitioners can use to inform care decisions. Still, such a clear typology is ancillary to our first aim, with basic descriptive analyses being sufficient for our purposes. Regardless of the means by which we do so, the ability to describe and summarize caregiver networks will support the second exploratory hypothesis positing that *Network Canvas will be an effective tool for mapping persons with memory loss–caregiver networks that researchers can adopt for future studies* (H2).

#### Aim 2: Determine Network-Based Predictors of Positive Well-Being for Caregivers and Persons With Memory Loss

In demonstrating the existence of caregiver networks and offering methods for measuring them, aim 1 will lay a strong foundation for additional analyses. The second aim is to start conducting these analyses, with a focus on identifying network-based predictors of positive well-being for both caregivers and persons with memory loss. The initial step in this effort will be testing another primary hypothesis, which states that the *caregiver support that persons with memory loss receive from their networks will be heterogeneous, with individual caregivers providing varying levels of support to a focal person with memory loss* (H3). Data collected during the name interpreting stage of the network survey will allow us to identify the forms of support focal persons with memory loss receive from specific caregivers, as well as the amount of influence each caregiver has in the life of their respective focal person with memory loss. Once again, descriptive statistics will offer an initial path to observing within-network heterogeneity in the quantity and quality of support provided by caregivers, as well as heterogeneity in caregiver influence. Assuming sufficient heterogeneity in these variables, subsequent analyses will use standard and ordinal logistic regression to identify factors predicting the amount of care and influence that caregivers give to a focal person with memory loss. We anticipate that caregiver age, education, employment status, relationship with the focal person with memory loss, and geographic distance from the focal person with memory loss will be among these factors.

Measures and insights used to test hypotheses 1 and 3 will undoubtedly find further use in our plans to test our final exploratory hypothesis, which posits that *negative network-based influences (eg, conflict, different expectations in care) and positive network-based influences (eg, teamwork, communication) will affect feelings of burden, anxiety, stress, and well-being of caregivers* (H4). Both quantitative and qualitative analyses to test this hypothesis will be conducted. Quantitatively, we will use multivariable linear and logistic regression to examine associations between Neuro-QoL Positive Affect and Well-Being measures and network-based measures, both for caregivers and persons with memory loss. Examples of network-based measures used will include the number of caregivers in an individual’s network, the number of other care recipients the caregiver is providing care for, closeness of relationships between caregivers, and the number of conflict relationships in the network. Anticipated control variables include personal factors of persons with memory loss (eg, cognition, comorbidities, health literacy, and self-efficacy), caregiver attributes, community-level measures, and participant societal perceptions (see *Interpersonal Network Measures* section for further details), accounting for all levels of our social ecological framework. When appropriate, we will examine within-network and between-network variance of outcome variables and perform multilevel analyses as needed.

Qualitatively, responses from open-ended questions in our instrument will be uploaded into NVivo 10 (Lumivero) for analysis. Responses will be analyzed using constant comparative techniques. Qualitative coders will independently assess responses for focal themes, then convene to compare and compile findings, creating a list of categories and major themes [71]. Identified themes will be discussed and refined through a series of coder meetings, during which coders will triangulate their perspectives and resolve any identified discrepancies through discussion [70]. We expect coders to identify several themes relevant to caregiver networks, conflict within these networks, and caregiver emotions, shedding light on important mechanisms underlying the effects of networks on caregiver well-being.

### Ethical Considerations

This study was registered and approved by the Northwestern University Institutional Review Board (STU00219675). All participants will complete online electronic informed consent prior to taking part in this study. Furthermore, all potential participants aged 65 years or older will be given the validated UBACC inventory to confirm their ability to provide informed consent [75]. Our team will only collect data from individuals deemed able to provide consent per the UBACC. Network data capture, as well as research on dyads of caregivers–people with dementia, routinely uses second-hand perceptions of study participants to understand their relationships with other individuals, particularly when the research imposes minimal risk and when it would be impossible to otherwise conduct.

During consent, participants will be informed that they can decline participation at any time. Participants who complete this study will receive a US $100 Target gift card as compensation. All study data will be stored only on secure servers hosted at Northwestern University, and only members of our research team will be able to access these data.

## Results

This study was funded in August 2023 by the National Institutes of Health (R01AG083034), with an expected end date of July 2028. The study protocol was approved by Northwestern University’s Institutional Review Board (STU00219675) and was piloted internally before participant enrollment began in January 2025. Recruitment began in January 2025, and as of March 2026, a total of 155 participants have been enrolled. Enrollment is planned to end by April 2027, with results expected between August and November 2027.

## Discussion

We expect our analyses to find that both persons with memory loss and their caregivers name multiple individuals as sources of support for persons with memory loss, confirming the existence of caregiver networks and their role in person with memory loss care (H1). Furthermore, individual caregivers within these networks are expected to vary in the types and amounts of support they provide to persons with memory loss (H3). This study is designed such that simple descriptive statistics derived from our data will sufficiently test these primary hypotheses. Additionally, we expect subsequent quantitative and qualitative analyses to reveal that network-based influences relate to self-reported feelings of burden, anxiety, stress, and well-being among surveyed caregivers (H3). These analyses will help identify potential mechanisms underlying the relationship between caregiver networks and the experiences of individual caregivers that future work can investigate. This work will demonstrate that Network Canvas is an effective tool for measuring and mapping caregiver networks of persons with memory loss, thereby paving the way for future research on these networks (H2).

As a whole, this study advances research on caregivers of persons with memory loss by operationalizing a scalable and replicable approach to measuring caregiver networks. The ability to easily measure and identify structural features of caregiver systems will help identify predictors of caregiver experiences and outcomes. Additionally, this study is expected to benefit the caregiver–person with memory loss research community. We intend to disseminate research findings through various channels that best meet the needs of our diverse audiences and stakeholders. Our initial dissemination efforts will take the form of academic research publications as well as presentations at conferences for both academic and caregiver stakeholder audiences.

Beyond publications, we intend to make parts of our instrument—in particular our Network Canvas component—widely available so that other researchers can conduct their own studies of caregiver networks. These resources will be accompanied by workshops that will help researchers adopt best practices and use the results of this study as an illustrative example.

Despite its contributions and innovations, this study is admittedly not without limitations. Given that participant data will only be captured at a single time point, this study will not be able to perform longitudinal analyses that can better support causal claims. Additionally, data for each of the sampled networks may not always come from the same number or kinds of participants. Some networks may be reported only by a focal person with memory loss, others only by a single caregiver, and still others by both a focal person with memory loss and multiple caregivers. Such variation in the sources of data will likely appear in networks of persons with memory loss with stronger signs of dementia, who will be unable to provide consent to participate in this study per the UBACC. Although this variation is necessary for feasible and ethical data collection, it may exclude important perspectives, particularly those of persons with memory loss with higher levels of cognitive impairment. While these are noteworthy limitations, we do not expect them to detract from the quality of insights gained from this study, nor the ability to map the entire system of support around persons with memory loss. Moreover, the methodological resources developed through this study will make it much easier for researchers to build new studies on caregiver networks of persons with memory loss, affording them time to devise new ways to address current limitations.

## Supplementary material

10.2196/98270 Peer Review Report 1 Peer-review report by the Center for Scientific Review Special Emphasis Panel RFA: Measures and Methods for Research on Family Caregivers for People Living with Alzheimer’s Disease and Related Dementias (AD/ADRD) (National Institutes of Health, USA).

## Abbreviations

AD: Alzheimer disease
AD8TM: Ascertain Dementia 8-item Informant Questionnaire
ADRD: Alzheimer disease–related dementia
LEAF: Life Enhancing Activities for Family Caregivers
NDR: Negotiations and Dispute Resolution
NIH: National Institutes of Health
PROMIS: Patient-Reported Outcomes Measurement Information System
REDCap: Research Electronic Data Capture
UBACC: University of California, San Diego Brief Assessment of Capacity to Consent
