# Supplementary material for: Caregiver Networks of Older Adults With Alzheimer Disease: Design and Protocol for a Multisite Study Using Network Canvas
Source: JMIR Res Protoc. 2026 Jul 13;15:e98270. doi: 10.2196/98270 (PMC13361618; doi:10.2196/98270)
Supplement: Peer Review Report 1 [file resprot-v15-e98270-s001.pdf]

SUMMARY STATEMENT
(Privileged Communication)

PROGRAM CONTACT:
AMELIA Karraker
301.555.1212
karrakeraw@nih.gov

Release Date: 03/30/2023
Revised Date:

Application Number: 1 R01 AG083034-01

Principal Investigators (Listed Alphabetically):

BIRKETT, MICHELLE
LINDQUIST, LEE A (Contact)

Applicant Organization: NORTHWESTERN UNIVERSITY AT CHICAGO

Review Group: ZRG1 AN-A (56)
Center for Scientific Review Special Emphasis Panel
RFA: Measures and Methods for Research on Family Caregivers for People Living with Alzheimer’s Disease and Related Dementias (AD/ADRD)

Meeting Date: 03/15/2023
Council: MAY 2023
Requested Start: 09/01/2023
RFA/PA: AG23-022
PCC: 2CDEMAK

Project Title: Measuring Caregiver Networks of Older Adults with Alzheimer's Disease

SRG Action: Impact Score:29
Next Steps: Visit https://grants.nih.gov/grants/next_steps.htm
Human Subjects: 30-Human subjects involved - Certified, no SRG concerns
Animal Subjects: 10-No live vertebrate animals involved for competing appl.
Gender: 1A-Both genders, scientifically acceptable
Minority: 1A-Minorities and non-minorities, scientifically acceptable
Age: 3A-No children included, scientifically acceptable

Project Year	Direct Costs Requested	Estimated Total Cost
1	499,750	788,559
2	499,965	788,899
3	499,277	787,813
4	498,354	786,357
5	499,761	788,577
TOTAL	2,497,107	3,940,204

1R01AG083034-01 LINDQUIST, LEE

RESUME AND SUMMARY OF DISCUSSION: The application proposes to measure the social network of caregivers of people living with Alzheimer's disease (PWD) and predictors of positive wellbeing using data from a multi-site observational study of PWD-caregiver networks. The investigators will use social network methodology software to measure PWD-caregiver networks. The panel noted that this examination of PWD-caregiver networks is a highly significant area of inquiry as less is known about caregiver networks for multiple family members and non-related caregivers compared to caregiver-PWD dyads. The findings may advance the understanding of caregiving systems to advance research among people living with Alzheimer's disease. The panel identified many additional key strengths of the application. The investigative team is strong as is the environment. The examination of prior research to support the study's significance for tiers of caregivers of PWD is rigorous. The examination of caregiving network tiers is rigorous and the use of the Network Canvas social network methodology software is innovative in this context. The study builds on prior work and the description of work with community partners is a strength of the application. The methods to capture the complexity of relationships and the level of care in the networks is rigorous as well as guided by an appropriate conceptual model. The attention to disease severity and the diversity among caregivers are major strengths of the approach. Minor weaknesses noted by the panel include unaddressed limitations of the convenience sample in the approach and the implications for the findings. Heterogeneity in the Latino population is not clearly addressed in the approach. Overall, the majority of the panel concurred that the application has potential for high scientific impact on the field and can advance the understanding of caregiving networks.

DESCRIPTION (provided by applicant): The overall goal of this proposal is to use a social network measurement methodology (Network Canvas) to capture expanded definitions of family caregivers related to people living with Alzheimer's disease and related dementias (AD/ADRD), with special focus on positive/negative relationship aspects. This proposed methodology will advance PWD-caregiver dyad research to encompass measuring real-world PWD-caregiver networks. Among people living with Alzheimer's disease (PWD), there is a significant population heterogeneity in how individuals define "family" caregivers. Most studies concentrate on the caregiver-PWD dyad with one singular primary caregiver. This dyad approach may not be represent real-life, since multiple family members and friends support the care of PWD. Multiple caregivers may also have differing ideas of obligation, expectations of care, and positive/negative aspects of care. Our prior research has shown that interactions between caregivers can be positive (e.g., teamwork to convince PWD to accept help) or negative (e.g., conflicts causing emotional distress). There is a lack of research on how these interpersonal interactions can be measured on a network level. To mitigate these relationship measurement gaps, our team has built a free, open-source, NIH funded software suite for researchers called Network Canvas, that identifies and maps social network data. We aim to: Aim 1. Measure and map the social network of caregivers of PWD using the Network Canvass methodology, to examine the population heterogeneity in how individuals define "family" caregivers. Aim 2. Measure network predictors of positive wellbeing for caregivers and PWD. We will conduct a large multi-site (IL, IN, HI) study of 200 PWD caregiver networks (up to 1000 people) - using snowball recruitment to identify/interview members of the networks – and the Network Canvas methodology. Recruitment will be through interdisciplinary community partners, physician offices, and ongoing NIH- funded cohorts - representing rural/urban, ethnic/racially diverse, LGBTQ groups. We anticipate multiple tiers of PWD-Caregiver networks: 1.) consentable PWD (mild, moderate AD) with caregivers, 2.) Caregivers only (PWD with severe dementia/unable to consent), 3.) consentable PWD only, no caregivers (un-befriended). Mixed method interviews will examine detailed patient-centered outcome measures, interpersonal interactions (positive/negative), and effects of the networks on the individual. Through this

research, we plan to have a 1.) Better understanding of how to measure caregiver networks, 2.) Identify which aspects of caregiver networks are most predictive of the impact on psychological and physical well-being of caregivers and PWD, and 3.) Operationalize a network measurement approach to understand caregiver systems that will be shareable with other researchers to provide larger benefit for the caregiver-PWD research community.

PUBLIC HEALTH RELEVANCE: Among people living with Alzheimer's disease (PWD)-caregiver research, most studies concentrate on the caregiver-PWD dyad which is not representative of real-life as multiple family members and non-related friends are often involved in the care of PWD. To advance the measurement of PWD-caregiver networks in research, we will use an NIH-funded Social Network methodology software (Network Canvas) in a multi-site observational study of 200 PWD-caregiver networks. Through this research, we plan to have a better understanding of how to measure PWD-caregiver networks and operationalize a network measurement approach to understand caregiver systems that will be shareable with other researchers.

CRITIQUE 1

Significance: 2
Investigator(s): 3
Innovation: 3
Approach: 3
Environment: 1

Overall Impact: This R01 proposal submitted by a research team (MPI) that seeks to use social network methodology (Network Canvas combined with surveys and open-ended questions) to develop a research measure of often complex AD/RD caregiver networks and communication links/supports. The proposal is responsive to RFA. Specifically, the proposal aims to gain understanding of the person with dementia (PWD)-caregiver networks, expand the definition of a network, identify aspects of a network predictive of PWD-caregiver well-being and conflict, and operationalize a network measurement approach for future research. The variables (measures) and socio-ecological model to be used are appropriate. Additionally, community support data items, perceptions of family obligations, and stigma associated with dementia are included in the data collection plan, which is a strength of the proposal. There is preliminary supportive research provided and Dr Lindquist has a track record of leading and conducting multi-site studies previously and Dr Birkett developed the Network Canvas software. Although, the 2 PIs appear not to have worked together previously. A weakness in the approach that creates some concern is that it is unclear with the recruitment sites and recruitment approach described that a sufficient number of diverse PWD (e.g., Persons with moderate to later stages of dementia, LGBTQ) will be recruited. This is a potential problem as the proposal aims to "expand caregiver definition to be inclusive" and subgroup analyses are planned to examine race ethnicity, cognitive impairment level, sexual orientation, and other group factors. In conclusion, the weaknesses are not major, and the proposed study may have moderate- high potential impact on the field of research focused on PWD -caregiver networks.

1. Significance:
Strengths

- Previous research included asking a series of open-ended questions (without the “ visual” Network canvas) to 97 family caregivers that found diverse caregiving networks beyond a dyadic (PWD-caregiver).
- The proposal seeks to address the lack of adequate measurement and expand understanding of diverse dementia caregiving networks which is needed for research and healthcare provider care planning.
- The use of the software (data collection and analysis) combined with data collection may have significant advantages over existing measurement approaches.

Weaknesses

- Little mention of previous research focused on assessment of caregiver networks.

2. Investigator(s):

Strengths

- Dr. Lindquist multiple previous research awards include those from AHRQ, NIH, and three from PCORI. Dr Lindquist has the established expertise in the research area, is a geriatrician, and leads the Northwestern University Claude Pepper Older American Independence Centers (OAIC) program that was awarded in 2020.
- Dr. Birkett is an expert in network data capture as demonstrated by her role as PI of the Network Canvas Software Development Grant (R01DA042711; MPIs: Birkett & Phillips).
- Several of the team members have collaborated (with the PIs) previously and there are a few publications.

Weaknesses

- It does not appear Drs Lindquist and Birkett have collaborated previously.
- This is an MPI proposal, the communication plan between the PIs and supervisory plan by the PI team is not well described.

3. Innovation:

Strengths

- The project is technically innovative by collecting and analyzing data with software that has been used in multiple previous NIH projects including assessing COVID-19 contact networks.
- What appears to be unique is Dr Birkett’s Network Canvas provides the opportunity to join with a sociogram, survey data to provide descriptors and calculate network composition (created by Network Canvas) and structure including the size, closeness, and support connections.

Weaknesses

- The use of a sociogram and snowballing to identify caregivers in itself is not unique and other approaches are not considered beyond Network Canvas.

4. Approach:

Strengths

- Interviews will be conducted in English and Spanish.
- The study population identified is appropriate (although Florida is identified as one of the geographic sites but there is no corresponding site description).
- The use conducting Zoom interviews has been established as a reliable approach in other populations.

Weaknesses

- There is limited discussion of previous research rigor and limitations focused on caregiving networks and how this approach specifically is advantageous.
- Other cognitive assessment measures (can be done via Zoom) that would provide severity of cognitive impairment which would be appropriate for this research assessment tool.
- Detail is missing re how the team “will specifically target low-socioeconomic individuals, LGTBQ, and racial-ethnic minorities” and how the PIs/co investigators will therefore obtain the targeted enrollment.
- It is unknown how nuances or idioms of key concepts across differing cultural groups will be addressed in research interviews.
- It is unknown if the identified measures are all available in Spanish. There is no consideration on how qualitative analysis will be done with data collected in Spanish.
- There are number of measures that PWD will be asked to complete but it is unknown if all of these measures have been established as reliable and valid in a moderately cognitively impaired population.

5. Environment:
Strengths
- Consortia on the proposal include Indiana University, Aging and In-Home Services of Northeast Indiana and First Vitals Health and Wellness.
- The NW University has the infrastructure to support the proposed study.

Weaknesses
- No score driving weaknesses.

Study Timeline:
Strengths
- Appropriate.

Weaknesses
- None noted.

Protections for Human Subjects:
Acceptable Risks and/or Adequate Protections
Data and Safety Monitoring Plan (Applicable for Clinical Trials Only):
Not Applicable (No Clinical Trials)

Inclusion Plans:
- Sex/Gender: Distribution justified scientifically
- Race/Ethnicity: Distribution justified scientifically
- For NIH-Defined Phase III trials, Plans for valid design and analysis: Not applicable
- Inclusion/Exclusion Based on Age: Distribution justified scientifically

Vertebrate Animals:
Not Applicable (No Vertebrate Animals)

Biohazards:

Not Applicable (No Biohazards)

Applications from Foreign Organizations:
Not Applicable (No Foreign Organizations)

Select Agents:
Not Applicable (No Select Agents)

Resource Sharing Plans:
Acceptable

Authentication of Key Biological and/or Chemical Resources:
Not Applicable (No Relevant Resources)

Budget and Period of Support:
Recommend as Requested

CRITIQUE 2

Significance: 2
Investigator(s): 1
Innovation: 2
Approach: 3
Environment: 1

Overall Impact: Co-PIs Drs. Lindquist and Birkett have proposed to measure caregiver networks in ADRD using an existing open software design developed for HIV populations by Dr. Birkett. The project builds upon Dr. Lindquist's expertise in dementia care and caregiving research coupled with Dr. Birkett's experience with network analysis. The study has clear achievable goals and importantly considers a range of ADRD caregiving scenarios and experiences and also focuses on positive and negative caregiving experiences. The team is well-qualified to recruit diverse samples and has experience working with their community partners.

1. Significance:
Strengths
- Limits of dyadic approach well-documented; need for more inclusive caregiver definition well-justified.
- This project nicely builds upon teams' preliminary research.

Weaknesses
- None noted.

2. Investigator(s):
Strengths

- Dr Lindquist has led multiple projects and teams; expertise in ADRD and caregiving research.
- Dr. Birkett has expertise in network analyses.
- Interdisciplinary collaboration that builds upon prior research of both investigators.

Weaknesses

- None noted.

3. Innovation:

Strengths

- Use of open-source social networking data – network canvas- will facilitate research beyond this study- impressive visual approach.
- Focus on positive and negative aspects of caregiving experience.

Weaknesses

- None noted.

4. Approach:

Strengths

- Inclusion of multiple types of ADRD caregiving networks (those with severe dementia, mild etc) important to capture variability in networks.
- Multisite recruitment including focus on LGBTQ populations.
- Working with community partners for recruitment efforts.
- Use of familialism scale is important dimension to include.

Weaknesses

- While focus on those without identifiable caregivers important, many ‘unbefriended’ (please consider other terminology that is less stigmatizing) may have POA or estranged family or non-traditional caregivers who the healthcare system does not usually identify-- additionally this group may themselves not be able to consent-- but could still be targeted for inclusion. It would be helpful to expand on how this group will be included.
- This is not nationally representative sample as it is described- it is a convenience sample targeted around PI’s contacts - justification for inclusion of specific cites (eg Hawaii needed) given limitation of 200 networks only.
- Limitation of 4 caregivers may limit knowledge-- those with larger networks may be misrepresented.

5. Environment:

Strengths

- Extensive resources available to support team.

Weaknesses

- None noted.

Protections for Human Subjects:

Acceptable Risks and/or Adequate Protections

Data and Safety Monitoring Plan (Applicable for Clinical Trials Only):

Acceptable

Protections for Human Subjects:
Acceptable Risks and/or Adequate Protections
Data and Safety Monitoring Plan (Applicable for Clinical Trials Only):
Not Applicable (No Clinical Trials)

Inclusion Plans:
- Sex/Gender: Distribution justified scientifically
- Race/Ethnicity: Distribution justified scientifically
- For NIH-Defined Phase III trials, Plans for valid design and analysis: Not applicable
- Inclusion/Exclusion Based on Age: Distribution justified scientifically

Vertebrate Animals:
Not Applicable (No Vertebrate Animals)

Biohazards:
Not Applicable (No Biohazards)

Applications from Foreign Organizations:
Not Applicable (No Foreign Organizations)

Select Agents:
Not Applicable (No Select Agents)

Resource Sharing Plans:
Acceptable

Authentication of Key Biological and/or Chemical Resources:
Not Applicable (No Relevant Resources)

Budget and Period of Support:
Recommend as Requested

CRITIQUE 3

Significance: 4
Investigator(s): 1
Innovation: 4
Approach: 3
Environment: 1

Overall Impact: This is an MPI collaboration between the creator of the Network Canvass social network tool and a dementia researcher. The project maps caregiver social network and assesses predictors of caregiver well-being. This information could be moderately useful to members of the care team going forward.

1. Significance:
Strengths
- Dementia caregiver research has focused on primary caregiver, this project would do a better job of collecting networks.

Weaknesses
- Aim 2 descriptive and probably does not need to happen.

2. Investigator(s):
Strengths
- Team developed the network canvas product, this project builds on their prior work.
- MPIs have complementary expertise.

Weaknesses
- New collaboration for the MPIs.

3. Innovation:
Strengths
- Prior work on network canvass was innovative.

Weaknesses
- Project itself not very innovate.

4. Approach:
Strengths
- Nice medley of caregivers targeted in recruitment.
- New application of Network Canvass project previously developed as NIH research.

Weaknesses
- Collecting many measures may burden respondents.
- Aim 2 is descriptive and unlikely to significantly extend the science.

5. Environment:
Strengths
- Consortia environment is appropriate.

Weaknesses
- None noted.

Protections for Human Subjects:
Acceptable Risks and/or Adequate Protections
Data and Safety Monitoring Plan (Applicable for Clinical Trials Only):
Not Applicable (No Clinical Trials)

Inclusion Plans:
- Sex/Gender: Distribution justified scientifically
- Race/Ethnicity: Distribution justified scientifically

- For NIH-Defined Phase III trials, Plans for valid design and analysis: Not applicable
- Inclusion/Exclusion Based on Age: Distribution justified scientifically

Vertebrate Animals:
Not Applicable (No Vertebrate Animals)

Biohazards:
Not Applicable (No Biohazards)

Applications from Foreign Organizations:
Not Applicable (No Foreign Organizations)

Select Agents:
Not Applicable (No Select Agents)

Resource Sharing Plans:
Acceptable

Authentication of Key Biological and/or Chemical Resources:
Not Applicable (No Relevant Resources)

Budget and Period of Support:
Recommend as Requested

THE FOLLOWING SECTIONS WERE PREPARED BY THE SCIENTIFIC REVIEW OFFICER TO SUMMARIZE THE OUTCOME OF DISCUSSIONS OF THE REVIEW COMMITTEE, OR REVIEWERS' WRITTEN CRITIQUES, ON THE FOLLOWING ISSUES:

PROTECTION OF HUMAN SUBJECTS: ACCEPTABLE

INCLUSION OF WOMEN PLAN: ACCEPTABLE

INCLUSION OF MINORITIES PLAN: ACCEPTABLE

INCLUSION ACROSS THE LIFESPAN: ACCEPTABLE

COMMITTEE BUDGET RECOMMENDATIONS: The budget was recommended as requested.

Footnotes for 1 R01 AG083034-01; PI Name: Lindquist, Lee A

NIH has modified its policy regarding the receipt of resubmissions (amended applications).See Guide Notice NOT-OD-18-197 at https://grants.nih.gov/grants/guide/notice-files/NOT-OD-18-

197.html. The impact/priority score is calculated after discussion of an application by averaging the overall scores (1-9) given by all voting reviewers on the committee and multiplying by 10. The criterion scores are submitted prior to the meeting by the individual reviewers assigned to an application, and are not discussed specifically at the review meeting or calculated into the overall impact score. Some applications also receive a percentile ranking. For details on the review process, see http://grants.nih.gov/grants/peer_review_process.htm#scoring.

MEETING ROSTER

Center for Scientific Review Special Emphasis Panel
CENTER FOR SCIENTIFIC REVIEW
RFA: Measures and Methods for Research on Family Caregivers for People Living with Alzheimer’s Disease and Related Dementias (AD/ADRD)
ZRG1 AN-A (56)
03/15/2023

Notice of NIH Policy to All Applicants: Meeting rosters are provided for information purposes only. Applicant investigators and institutional officials must not communicate directly with study section members about an application before or after the review. Failure to observe this policy will create a serious breach of integrity in the peer review process, and may lead to actions outlined in NOT-OD-22-044 at https://grants.nih.gov/grants/guide/notice-files/NOT-OD-22-044.html, including removal of the application from immediate review.

CHAIRPERSON(S)

FINGERMAN, KAREN L, PHD
PROFESSOR
DEPARTMENT OF HUMAN DEVELOPMENT & FAMILY SCIENCES
SCHOOL OF HUMAN ECOLOGY
THE UNIVERSITY OF TEXAS AT AUSTIN
AUSTIN, TX 78712

MEMBERS

AJROUCH, KRISTINE J, PHD
PROFESSOR
INSTITUTE FOR SOCIAL RESEARCH
UNIVERSITY OF MICHIGAN
ANN ARBOR, MI 48106

BROWN, ELLEN LESLIE, EDD
ASSOCIATE PROFESSOR
DEPARTMENT OF HEALTH SCIENCES
COLLEGE OF NURSING
FLORIDA INTERNATIONAL UNIVERSITY
MIAMI, FL 33199

EPSTEIN-LUBOW, GARY P, MD
ASSOCIATE PROFESSOR
DEPARTMENT OF PSYCHIATRY AND HUMAN BEHAVIOR
THE WARREN ALPERT MEDICAL SCHOOL
BROWN UNIVERSITY
PROVIDENCE, RI 02906

GRIFFIN, JOAN M, PHD
PROFESSOR
HEALTH SERVICES RESEARCH
COLLEGE OF MEDICINE
MAYO CLINIC
ROCHESTER, MN 55905

LEE, JUNG-AH, PHD
ASSOCIATE PROFESSOR
SUE & BILL GROSS SCHOOL OF NURSING
UNIVERSITY OF CALIFORNIA, IRVINE
IRVINE, CA 92697

LIN, I-FEN, PHD
PROFESSOR
DEPARTMENT OF SOCIOLOGY
BOWLING GREEN STATE UNIVERSITY
BOWLING GREEN, OH 43403

MAUSBACH, BRENT T, PHD
PROFESSOR IN-RESIDENCE
DEPARTMENT OF PSYCHIATRY
UNIVERSITY OF CALIFORNIA, SAN DIEGO
LA JOLLA, CA 92093

MITTELMAN, MARY SHERMAN, DRPH
RESEARCH PROFESSOR
DEPARTMENTS OF PSYCHIATRY AND REHABILITATIVE MEDIC
NEW YORK UNIVERSITY
NEW YORK, NY 10016

NICHOLAS, LAUREN HERSCH, PHD
ASSOCIATE PROFESSOR
ANSCHUTZ MEDICAL CAMPUS
ANSCHUTZ MEDICAL CAMPUS
UNIVERSITY OF COLORADO DENVER
AURORA, CO 80045

ORNSTEIN, KATHERINE A, PHD
DIRECTOR
CENTER FOR EQUITY IN AGING
SCHOOL OF NURSING
JOHNS HOPKINS UNIVERSITY
BALTIMORE, MD 21211

RODRIGUEZ, MIRIAM JOCELYN, PHD
ASSOCIATE PROFESSOR
SCHOOL OF MEDICINE
INDIANA UNIVERSITY BLOOMINGTON
BLOOMINGTON, IN 47405

SAVLA, JYOTI S, PHD
PROFESSOR
DEPARTMENT OF HUMAN DEVELOPMENT & FAMILY
SCIENCE
COLLEGE OF LIBERAL ARTS AND HUMAN SCIENCES
VIRGINIA POLYTECHNIC INSTITUTE & STATE UNIVERSITY
BLACKSBURG, VA 24061

VAN HOOK, JENNIFER, PHD
PROFESSOR
DEPARTMENT OF SOCIOLOGY
PENNSYLVANIA STATE UNIVERSITY
UNIVERSITY PARK, PA 16802

SCIENTIFIC REVIEW OFFICER

BELLINGER, JESSICA, PHD
SCIENTIFIC REVIEW ADMINISTRATOR
CENTER FOR SCIENTIFIC OF REVIEW
NATIONAL INSTITUTES OF HEALTH
BETHESDA, MD 20892

EXTRAMURAL SUPPORT ASSISTANT

HAUCK, CATHERINE BRYNN CAMPBELL
LEAD EXTRAMURAL SUPPORT ASSISTANT
CENTER FOR SCIENTIFIC REVIEW
NATIONAL INSTITUTES OF HEALTH
BETHESDA, MD 20892

KELLY, COURTNEY LYNN
EXTRAMURAL SUPPORT ASSISTANT
CENTER FOR SCIENTIFIC REVIEW
NATIONAL INSTITUTES OF HEALTH
BETHESDA, MD 20892

Consultants are required to absent themselves from the room during the review of any application if their presence would constitute or appear to constitute a conflict of interest.
